# Can American College of Radiology in-training examination scores be used to predict Canadian radiology licensing examination results? A respective study

**DOI:** 10.1186/1472-6920-13-17

**Published:** 2013-02-06

**Authors:** Trent H Orton, Matthew McInnes

**Affiliations:** 1 Department of Medical Imaging at the Ottawa Hospital, University of Ottawa Faculty of Medicine, Room C159, The Ottawa Hospital Civic Campus, 1053 Carling Avenue, Ottawa K1Y 4E9, Canada; 2 Department of Medical Imaging at the Ottawa Hospital/ The Ottawa Hospital Research Institute, University of Ottawa Faculty of Medicine, Room C159, The Ottawa Hospital Civic Campus, 1053 Carling Avenue, Ottawa K1Y 4E9, Canada

**Keywords:** In-training examination, Licensing examination, Residency, Radiology

## Abstract

**Background:**

The purpose of this study is to evaluate the relationship between American College of Radiology (ACR) in-training examination scores and performance on the Royal College of Physicians and Surgeons of Canada (RCPSC) radiology licensing examination.

**Methods:**

Percentile ACR examination scores for 67 residents were obtained from 1995 to 2011 for four years of training and compared with results of the RCPSC examination. Mean ACR scores of residents who passed and residents who failed their RCPSC examination were compared with a t-test. ACR scores and licensing examination results were correlated. Logistic regression was used to predict the probability of failure given an individual’s ACR score. Receiver Operating Characteristic (ROC) curves were developed in order to estimate a threshold ACR score at or above which the risk of failure was negligible.

**Results:**

The ACR scores between residents who passed their licensing exam and those who failed were significantly different. There was moderate correlation between ACR scores and exam results. Using ROC curves for each year of training, the threshold ACR scores at or above which there was a negligible risk of exam failure were 32, 42, 63, and 47 for training years 1, 2, 3 and 4 respectively. Logistic regression curves, with 95% confidence intervals, were plotted for each year of training to predict RCPSC exam results based on an individual’s ACR score.

**Conclusions:**

ACR exam scores are a strong predictor of RCPSC examination performance. Percentile ACR scores can be used to identify residents at risk for future examination failure.

## Background

Canadian radiology residents are licenced to practice radiology through the administration of the Royal College of Physicians and Surgeons of Canada (RCPSC) radiology board examination at the end of their four years of radiology specific training. This examination consists of three parts: a written multiple-choice exam, an objective structured clinical examination (OSCE) and an oral examination. All three components must be passed to qualify to practice radiology in Canada.

One of the crucial roles of a radiology residency program director is to identify residents within their program at risk for unsuccessful completion of the RCPSC board examination. Identifying those at risk of failure well in advance of their examination allows residents and program directors the time and stimulus to make any necessary training adjustments. There are few objective measures that may identify a resident at risk. One such tool is the American College of Radiology (ACR) in-training examination. This is a multiple choice examination conducted annually near the end of each of the four years of radiology-specific training in those radiology programs that choose to administer it. The resident obtains a percentile ranking based on the performance of all residents writing the exam according to their year of training.

Resident ACR in-training examination scores have previously been shown to correlate significantly with American Board of Radiology (ABR) written exam results both in single institutional [1] and multi-institutional [2] trials. However, to our knowledge, no study has examined the relationship between ACR in-training scores and RCPSC radiology exam results. Further, no study has demonstrated threshold ACR scores for each year of training above which the risk of board examination failure becomes negligible. Finally, no model has yet been developed to help predict an individual resident’s risk of failure based on their individual ACR score.

We sought to evaluate the relationship between in-training ACR results and the RCPSC radiology licensing examination. The objective of this study was to test the null hypothesis that there is no significant difference between ACR in-training scores in those residents that passed and those that failed their licensing examination.

If our hypothesis was confirmed and there was indeed a difference between ACR scores in those that passed and those that failed their exams, we then sought to determine if a threshold ACR score could be estimated within each year of training above which there was a negligible risk of future examination failure. Additionally, we looked to develop a model to predict an individuals’ risk of failure based on their year of training and ACR score.

## Methods

Ethics approval was obtained from the Ottawa Hospital Research Ethics Board. Percentile scores of the American College of Radiology (ACR) in-training examination were obtained for 67 residents during a 17-year period (1995–2011) over all 4 years of radiology training at the University of Ottawa [post graduate years (PGY) 2 to 5]. Results for the first attempt at the RCPSC diagnostic radiology exam results were also collected for all 67 residents. The Royal College only provides residents and residency programs with pass/fail dichotomous results. The absolute scores are not made available.

The residents were separated into two groups, those who passed their Royal College examination on their first attempt and those who failed. The mean of the ACR in-training examination scores at each year of training were compared between both groups with an independent sample t-test, supplemented with a Levene’s test to assess for variance equality. The strength and direction of correlation between ACR examination scores and exam result was assessed with a biserial correlation coefficient, as the dichotomous designation of passing or failing the RCPSC exam is an artificial one defined by the college examiners [3]. An average ACR score was calculated for each resident’s four years of radiology training and correlated with RCPSC exam results. Furthermore, the ACR scores of each resident were correlated with their ACR scores the following year over the four years of radiology-specific training using a Pearson correlation coefficient. This was used as an assessment of the consistency of performance of residents over time.

Receiver operating characteristic (ROC) curves were plotted for each year of training. In identifying a threshold ACR score at which point there is a negligible risk of failure, we chose to minimize the false positive rate. That is, we chose to minimize the proportion of residents who failed their RCPSC examination that had ACR scores higher than the chosen threshold.

As the dependent variable, RCPSC exam result, is dichotomous, logistic regression was used to develop a model to predict RCPSC exam result based on the value of an individual’s ACR score.

Data analysis was performed with SPSS Statistics 19 (International Business Machines, Somers NY) and SAS statistical software (SAS Cary NC).

## Results

Annual American College of Radiology (ACR) in-training examination scores of residents at our institution who had taken their Royal College of Physicians and Surgeons of Canada (RCPSC) radiology licensing examination were obtained over a 17 year period from 1995 to 2011. Over this time period, 67 residents had taken the RCPSC licensing exam. 10 of these 67 residents (15%) failed their licensing examination on their first attempt.

ACR Scores for the first year of radiology training were not available for 9 residents. Two scores were not available for the second year of training. Ten scores were not available for the third year of training and 12 scores were not available for the fourth and final year of training. Two hundred and thirty five of 268 possible total ACR scores were available for analysis. RCPSC exam results on first attempt were available for all 67 residents.

Table 1 shows the comparison of ACR scores of those residents who failed their licensing exam and those who passed. There was a statistically significant difference between ACR scores of both groups in all 4 years of training (Table 1; column 4). The mean ACR scores were significantly higher in the group that passed versus the group that failed in all four years, with a difference between the means ranging from an ACR score difference of 29.321±16.651 to 36.957±12.914. Although the greatest difference in means was in PGY 5, there was no consistent trend toward a greater difference in means with further training.

**Table 1:** Mean ACR score difference and correlation between ACR scores and RCPSC exam results between those that passed and those that failed their RCPSC exams (CI = Confidence Intervals)

Year of training*	**RCPSC exam result**	**N†**	**Mean ACR score [95% CI]**	**Mean ACR difference [95% CI]**	**Biserial r**_**b**_
PGY2	Passed	50	46.08 [37.97, 54.19]	34.33 [23.75, 44.91] (P<0.0005) ‡	0.640
	Failed	8	11.75 [3.99, 19.51]		
PGY3	Passed	56	51.48 [43.47, 59.49]	35.70 [24.33, 47.07] (P<0.0005)	0.635
	Failed	9	15.78 [6.86, 24.70]		
PGY4	Passed	49	56.57 [48.59, 64.55]	29.32 [12.67, 45.97] (P=0.002)	0.563
	Failed	8	27.25 [11.67, 42.83]		
PGY5	Passed	46	54.46 [45.67, 63.24]	36.96 [24.04, 49.87] (P<0.0005)	0.661
	Failed	8	17.50 [6.93, 28.07]		
Average	Passed	57	51.42 [44.60, 58.24]	33.75 [24.42, 43.08] (P<0.0005)	0.692
	Failed	10	17.67 [10.68, 24.65]		

RCPSC = Royal College of Physicians and Surgeons of Canada, ACR = American College of Radiology, CI = confidence interval, PGY = post graduate year.

*PGY2 is the first year of 4 consecutive years of radiology-specific specialty training.

†Missing ACR values were omitted from analysis therefore the sample size varied in each year.

‡ Statistical significance at P ≤ 0.05.

Table 1 also summarizes the biserial correlation coefficients for all training years as well as the mean ACR scores for the groups who passed or failed their RCPSC examination on their first attempt (scores by PGY year and overall mean provided). ACR scores were found to correlate in a strongly positive manner with RCPSC licensing exam results in all four years. Although the highest training year correlation was in the final year of training, there was no consistent trend of increasing correlation with further training. Exam result correlation was strongest for the average ACR score. There was strong positive correlation between the ACR scores of consecutive training years with Pearson correlation coefficients of 0.774, 0.733 and 0.663 comparing PGY 2 and 3, PGY 3 and 4 and PGY 4 and 5 respectively.

**Figure 1:**
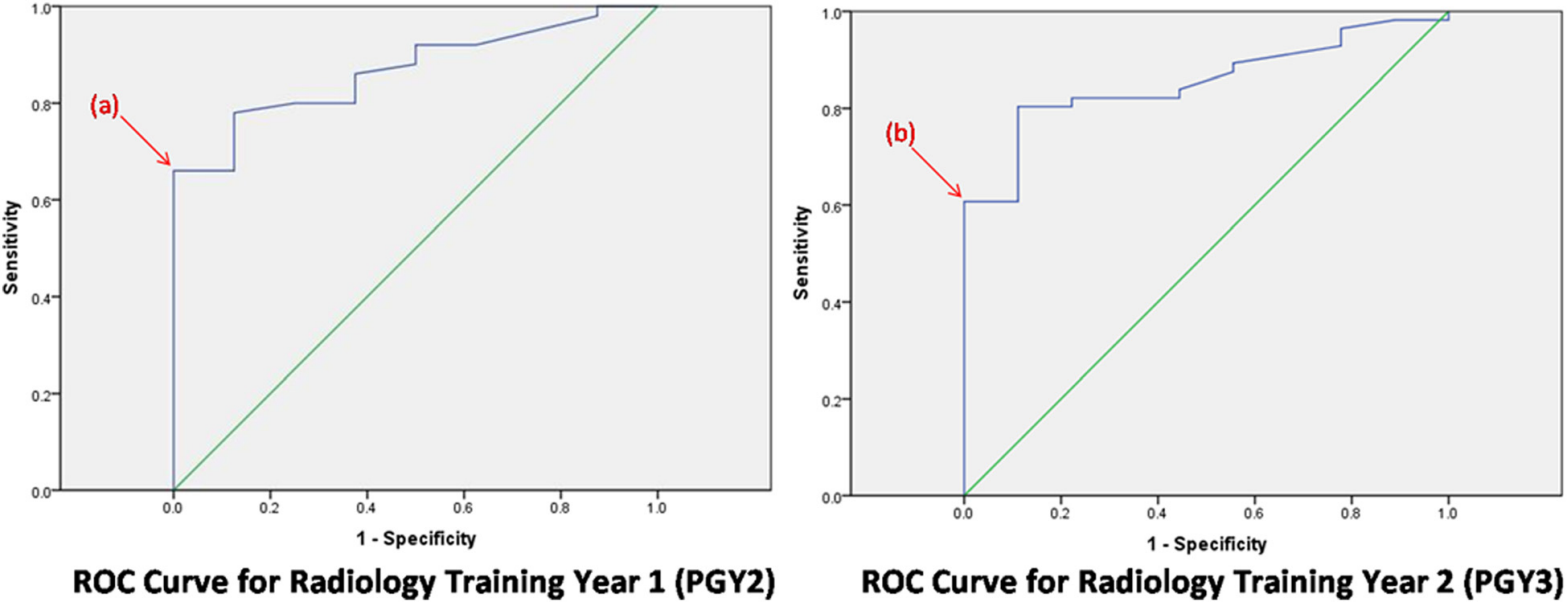
**In selecting a threshold American College of Radiology (ACR) score, we chose to minimize the proportion of failed students with ACR scores above the threshold (ie minimize false positive rate or 1 – Specificity).** In PGY 2, the maximum sensitivity (proportion of residents with ACR scores above the threshold who passed their exam or true positive rate) at this false positive rate occurred at point (a). This correlated to an ACR threshold percentile score of 32. In PGY 3, the maximum sensitivity occurred at point (b), which correlated to a percentile score of 42.

**Figure 2:**
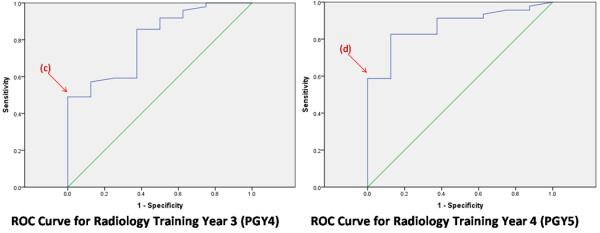
**In selecting a threshold American College of Radiology (ACR) score, we chose to minimize the proportion of failed students with ACR scores above the threshold (ie minimize false positive rate or 1 – Specificity).** In PGY 4, the maximum sensitivity (proportion of residents with ACR scores above the threshold who passed their exam, or true positive rate) at this false positive rate occurred at point (c). This correlated to an ACR threshold percentile score of 63. In PGY 5, the maximum sensitivity occurred at point (d), which correlated to a percentile score of 47.

Figures 1 and 2 show the ROC curves for each year of training. In selecting our threshold ACR score for each year of training at or above which there was a negligible risk of failure, we chose to minimize the proportion of failed students with ACR scores above the threshold. That is, we chose to minimize the false positive rate or 1 – Specificity. The subsequent threshold ACR scores are summarized in Table 2, column 2.

**Table 2:** Data generated from ROC curves and logistic regression

Year of training*	**Threshold ACR score†**	**OR‡**	**95% CI for OR**	**100 × [OR −1] (%) §**	ACR 50**	**95% CI for ACR 50**
PGY2	32	0.91 (P=0.0068)	[0.84, 0.97]	−9.3	4	[0, 13]
PGY3	42	0.93 (P=0.0014)	[0.89, 0.97]	−6.8	4	[0, 15]
PGY4	63	0.94 (P=0.0091)	[0.90, 0.98]	−6.0	8	[0, 23]
PGY5	47	0.93 (P=0.0039)	[0.88, 0.98]	−7.3	8	[0, 20]

PGY=post graduate year, ACR=American college of radiology, OR=odds ratio, CI=confidence interval.

* PGY2 is the first year of 4 consecutive years of radiology-specific specialty training.

† Threshold ACR score at or above which point there is a negligible risk of exam failure. Estimated from the ROC curves.

‡ Odds ratios were calculated using logistic regression. Statistical significance at P ≤ 0.05.

§ 100 × [OR-1] is a formula that gives the percentage change in the odds of failing for every 1 unit increase in the ACR score. A negative value, as in this case, indicates the odds of failing decrease with increases in the ACR score.

** ACR 50 is the ACR score at which point there was a 50% chance of failure. This was estimated for each year of training using the logistic regression curves.

**Figure 3:**
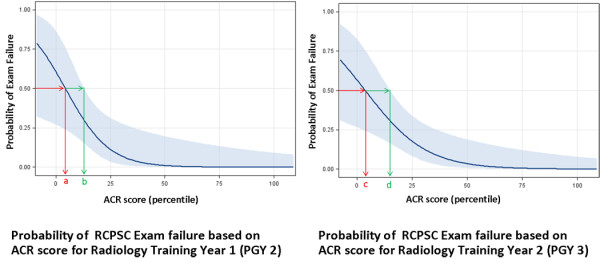
**The 95% confidence interval (CI) is within the light blue shaded region.** For PGY 2, the ACR score at which point there was a 50% chance of failure was approximately 4 (point a). The upper limit of the 95% CI was 13 (point b). For PGY 3, the ACR score at which point there was a 50% chance of failure was 4 (point c) and the upper limit of the 95% CI was 15 (point d).

**Figure 4:**
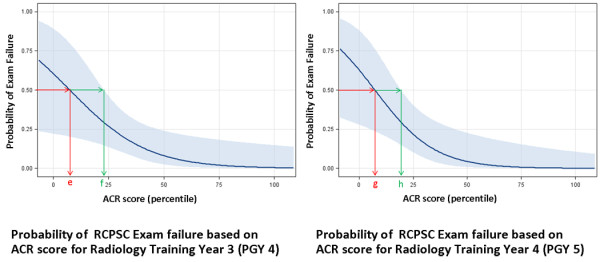
**The 95% confidence interval (CI) is within the light blue shaded region.** For PGY 4, the ACR score at which point there was a 50% chance of failure was approximately 8 (point e). The upper limit of the 95% CI was 23 (point f). For PGY 5, the ACR score at which point there was a 50% chance of failure was 8 (point g) and the upper limit of the 95% CI was 20 (point h).

**Figure 5:**
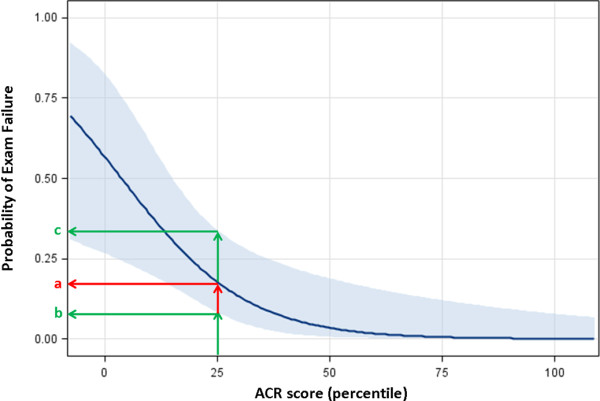
**The 95% confidence interval (CI) is within the light blue shaded region.** These models allow a program director to estimate a resident’s risk (PGY 3) of RCPSC examination failure based on their year of training and ACR score. For example, a resident scoring in the 25^th^ percentile has an 18% chance of failure (a) with a 95% confidence interval (CI) lower limit of 8% (b) and a 95% CI upper limit of 32% (c).

Figures 3 and 4 show the probability of licensing examination failure based on a resident’s ACR score for each year of training. These graphs were developed using logistic regression. There is a non-linear inverse relationship between the probability of examination failure and ACR score. As a resident’s ACR score increases their probability of failing their licensing examination decreases. These graphs can be used to estimate an individual residents risk of failure based on their individual ACR score (see Figure 5).

Figures 3 and 4 demonstrate the ACR score in each year of radiology training at which point there is a 50% chance of failure (ACR 50). These values are summarized in Table 2, column 6. There was a general trend towards increasing ACR 50 values with increased levels of training.

Logistic regression was used to calculate odds ratios with 95% confidence intervals which are summarized in Table 2, column 3 and 4. The odds ratios ranged from 0.91 in PGY 2 to 0.94 in PGY 4. The odds ratios can be interpreted as follows: for PGY2, residents that achieved one percentile score higher have 0.91 times the probability of failing their RCPSC exam compared to residents that had one score lower. A more convenient way to think of this is by using the formula 100 × (odds ratio – 1) which gives the percentage change in the odds of failing for every 1 unit increase in the ACR score. For example, for PGY 2, 100 × (odds ratio – 1) = −9.3%. Therefore, in PGY 2, the odds of failing the RCPSC exam decreased 9.3% for every 1 score increase in the ACR score.

## Discussion

The American College of Radiology (ACR) in-training examination is broadly used as an objective measure of radiology resident performance in North America. This examination provides feedback to the resident and program director and assesses their progress in preparation for their end of training examination.

Previous studies have identified the ACR score as an effective predictor of future written board performance [1,2]. Our study confirms that a similar relationship exists with the RCPSC diagnostic radiology exam. The mean ACR scores of residents who passed their RCPSC radiology board exam were significantly higher than for those residents who failed. There was strong correlation between ACR scores and board exam results. The odds ratios in all four years of training were less than 1 and statistically significant; therefore, higher ACR scores were associated with a reduced probability of board examination failure.

Not surprisingly, the average ACR score was the best predictor of exam failure when compared to ACR scores of each year of training. It showed the highest correlation with RCPSC exam result. This result was consistent with prior studies [1,2]. Baumgartner and Brothers Peterman attributed these results to the reduction in variability related to the resident taking the in-training examination multiple times.

Additionally, residents’ ACR scores correlated with each other in a strongly positive manner in consecutive years. This indicated that those residents who performed poorly on their ACR examination early in their training also tended to perform poorly on subsequent ACR exams throughout their training.

After confirming the utility of the ACR in-training examination as a future predictor of board examination performance, our objective was to then provide program directors with useful tools to give residents feedback on their current knowledge and study habits. We used receiver operator characteristic (ROC) curves and logistic regression for this purpose.

The concept of using receiver operator characteristic (ROC) curves for prediction of exam performance was used previously in a similar study performed by our anaesthesia colleagues [4]. However, we used these curves in a slightly different manner. We chose to minimize the risk of failing the exam with a high ACR score (false positive rate), thereby creating a novel threshold ACR score above which there was a negligible risk of failing the board exam. Our threshold scores ranged from the 32^nd^ to the 63^rd^ percentile. These ACR scores were higher than the previously reported ACR scores of 20^th^ percentile or less which predicted poor board performance [1,2]. This was not surprising as we were measuring a different parameter. We wanted program directors to be able to provide residents a realistic goal ACR score to attempt to surpass in each year of their training. This score would not just be predictive of better than poor performance on boards, but would (at least in theory) essentially eliminate the risk of board exam failure.

Logistic regression allowed us to provide program directors and radiology residents with three more pieces of information to chart training progress. Firstly, we calculated odds ratios for each year of training and used the formula: 100 × (odds ratio – 1) to provide residents with the percentage reduction in the odds of failing for each additional percentile point achieved.

We then used logistic regression curves to plot the probability of board exam failure versus the ACR percentile score. These curves allowed estimation of the ACR score at which point there was a 50% risk of RCPSC radiology board examination failure (ACR 50). These values are surprisingly low, ranging from the 4^th^ to 8^th^ percentile. However, including the upper limit of the 95% confidence interval as a more conservative measure, places the range from the 13^th^ to 23^rd^ percentile depending on the year of training. This more conservative number could be considered a risk of “poor” board exam performance. Interestingly, this ACR score range is similar to the prior American data in which a poor or failing board exam performance was seen in the ACR score range of less than 20 [1,2].

Finally, the logistic regression curves have been provided as easy-to-use board exam prediction models for each year of training. Each resident and program director can estimate their risk of examination failure (with 95% confidence intervals) based on their ACR score. It should be noted that the majority of the reduction in the probability of examination failure occurs up to an ACR score of approximately 50 (the steep portion of the curve in all four years of training).

The limitations of this study may include applicability to other radiology programs with different demographics. We are a medium-sized radiology program of six to eight residents per year, including one to two international medical graduate positions. We likely place more emphasis on certain aspects of our training than other programs. This limitation could be addressed with a multi-centre study involving multiple Canadian radiology residency programs.

Additionally, we are limited by the data provided to us from the College regarding exam performance. Residents and residency programs are provided simply with a pass or fail result for the entire examination. Absolute scores are not provided, nor are the examination results (pass/fail) for the individual components (multiple-choice, OSCE and oral exams). The ACR examination is written in a multiple-choice format and presumably would be a better predictor of the multiple-choice component of the boards than the other two. Unfortunately, we are unable to test this hypothesis.

Predicting future exam performance is fraught with uncertainty. Countless confounders can and do make certain success far less certain. This is particularly true with the oral exam component of the RCPSC radiology examination, which places additional emphasis on ‘exams-manship’. However, despite these limitations, the ACR exam has been shown to be a useful predictor of exam performance [1,2]. Our study agreed with these findings. In addition, we have developed several tools we hope will help radiology residents with self-assessment, and enable program directors to better guide their residents in their board examination preparation.

## Conclusions

ACR exam scores are a strong predictor of RCPSC examination performance. Percentile ACR scores can be used to identify residents at risk for future examination failure.

## Abbreviations

ACR: American College of Radiology; RCPSC: Royal College of Physicians and Surgeons of Canada; ABR: American Board of Radiology; ROC: Receiver Operating Characteristic; PGY: Postgraduate Year.

## Competing interests

No financial or non-financial competing interests for either author.

## Authors’ contributions

MM provided the idea and framework for the study and edited the manuscript. TO performed the data analysis, generated the figures and wrote the primary manuscript draft. Both authors read and approved the final manuscript.

## Authors’ information

MM is the diagnostic radiology residency program director at the University of Ottawa, and is a member of the RCPSC Diagnostic Radiology Specialty Committee as well as the Canadian Association of Radiology Program Directors. TO is a resident in diagnostic radiology at the University of Ottawa.

## Pre-publication history

The pre-publication history for this paper can be accessed here:

http://www.biomedcentral.com/1472-6920/13/17/prepub

## References

[B1] Baumgartner BR Brothers Peterman S Relationship between American College of Radiology In-training Examination Scores and American Board of Radiology Written Examination Scores Acad Radiol 1996 3 873 878 10.1016/S1076-6332(96)80281-9 8923908

[B2] Baumgartner BR Brothers Peterman S Relationship between American College of Radiology In-training Examination Scores and American Board of Radiology Written Examination Scores. Part 2. Multi-institutional Study Acad Radiol 1998 5 374 379 10.1016/S1076-6332(98)80156-6 9597105

[B3] Howell DC Statistical Methods for Psychology 1997 4 Wadsworth Publishing Company, Belmont

[B4] Kearney RA Sullivan P Skakun E Performance on ABA-ASA In-training Examination predicts success for RCPSC certification Can J Anesth 2000 47 9 914 918 10.1007/BF03019676 10989866

